# Electrical Remodeling of Preoptic GABAergic Neurons Involves the Kv1.5 Subunit

**DOI:** 10.1371/journal.pone.0096643

**Published:** 2014-05-05

**Authors:** Iustin V. Tabarean

**Affiliations:** The Department of Molecular and Cellular Neuroscience, The Scripps Research Institute, La Jolla, California, United States of America; University of Iowa, United States of America

## Abstract

The electrogenic machinery of an excitable cell can adapt in response to changes in input, genetic deficit or in pathological conditions, however the underlying molecular mechanisms are not understood. In cases of genetic deletion it is commonly observed that a channel subunit from the same family replaces the missing one. We have previously reported that Kv4.2−/− preoptic GABAergic neurons display identical firing characteristics to those of wild-type neurons despite having reduced A-type currents, and that, surprisingly, they present a robust upregulation of a delayed rectifier current, the nature of which is unknown. Here, using pharmacology, qPCR and Western blots we report that, although the wild-type neurons express several Kv subunits, the upregulated current is conducted by the Kv1.5 subunit exclusively. Thus, this study reveals the molecular nature of a novel mechanism of electrical remodeling in central neurons.

## Introduction

Excitable cells can remodel the complement of voltage-gated channel they express in response to changes in physiological input or in pathological conditions [1]–[4]. Thus, magnocellular neurosecretory cells of the supraoptic hypothalamic neurons increase the expression of specific sodium channel subunits in response to different osmotic milieus [3]. Electrical remodeling of the heart, which predisposes to arrhythmias, is an example of a pathological adaption in the electrogenic machinery of an excitable cell [1]. In situations of genetic deletion of a Nav subunit it is commonly observed a compensatory increase in the expression of a subunit from the same family, although the precise mechanism may be cell-specific. For instance, in mice lacking Nav1.6, the Nav1.1 is upregulated in Purkinje neurons whereas Nav1.2 is upregulated in retinal ganglion cells [3], [5]. The molecular mechanisms involved in electrical remodeling of excitable cells are not understood.

The tonic firing activity of preoptic GABAergic neurons plays an important role in the control of thermoregulatory networks (reviewed in [6]). We have previously reported that the spontaneous firing activity of these neurons is potently affected by changes in the amplitude of the A-type K current, and that Kv4.2 and Kv4.3 subunits are expressed in the wild-type (w-t) neurons [7]. Surprisingly, Kv4.2−/− GABAergic preoptic neurons present identical firing properties with w-t neurons although they have reduced A-type currents (in spite of compensatory upregulation of the Kv4.1 subunit) [7]. However, these neurons also present a robust upregulation in the delayed rectifier current (I_DR_) [7]. In order to gain further insights in the mechanisms of electrical remodeling in preoptic neurons, in this study we have determined the molecular nature and the electrophysiological properties of the increased I_DR_ of Kv4.2−/− GABAergic preoptic neurons to test the hypotheses that the upregulation involves a specific Kv channel subunit and that it is responsible for the conserved firing characteristics of Kv4.2−/− neurons.

## Materials and Methods

### Ethics statement

All animal work was conducted in accordance with the Institutional Animal Care and Use Committee of the Scripps Research Institute (approval ID #08-0129). The standards are set forth by American Association for the Accreditation of Laboratory Animal Care (AAALAC) standards and the regulations set forth in the Animal Welfare Act.

### Animals and slice preparation

The Kv4.2−/− mice were a kind gift from Dr Jeanne Nerbonne (Washington University, St. Louis). The GAD65-GFP mice were a kind gift from Dr Gabor Szabo (Hungarian Academy of Sciences, Budapest, Hungary). Both transgenic lines are on the C57/Bl6 background. We have crossed the two lines to obtain double transgenic Kv4.2−/− GAD65-GFP mice. Genotyping of littermates was performed by PCR using Kv4.2-specific primers as described by others [8]. Neonatal mice were also phenotyped by the observation of GFP fluorescence in the intact brains of mice exposed to blue light illumination. In this studywe refer to double transgenics as “Kv4.2−/−;GAD65GFP”. Coronal tissue slices (250 µM thick) containing the median preoptic nucleus (MnPO) were prepared from GAD65-GFP or Kv4.2−/−;GAD65-GFP male mice (28–42 days old) as described previously [9]. The slice used in our recordings corresponded to the sections located from 0.5 mm to 0.25 mm from bregma in the mouse brain atlas [10]. The slices were prepared at 9–11 am local time.

### Dissociated preoptic neurons from slices

To allow a faster exchange of extracellular solutions and to avoid space clamp limitations as well as possible presynaptic effects activated by pharmacological treatments we have carried out this study on acutely dissociated preoptic neurons from slices. The MnPO was punched out of a brain slice and incubated in Hibernate A and papain (1 mg/ml) for 10 min. After washing out the enzyme with Hibernate –A the cells were dissociated by gentle trituration with a fire-polished Pasteur pipette. The cell suspension was pelleted (1000 xg for 2 min) and resuspended in Neurobasal medium and then plated on coverslips (Biocoat, BD Biosciences, Belgium). Cells were allowed to attach to the coverslips for 2–3 hours before recording. GABAergic preoptic neurons were identified using the GFP-fluorescent signal under 480 nm wavelength excitation. Cultures were used for recordings for up to 10 hours after dissociation.

### Whole-cell patch clamp recording

The artificial cerebrospinal fluid (aCSF) contained (in mM): 130 NaCI, 3.5 KCI, 1.25 NaH_2_PO_4_, 24 NaHCO_3_, 2 CaCI_2_, 1 MgSO_4_, 10 glucose osmolarity of 300–305 mOsm, equilibrated with 95% O_2_ and 5% CO_2_ (pH 7.4). Other agents were added to this medium. In some experiments the aCSF was supplemented with TTX (1 µM) to block voltage-gated Na^+^ currents. A K^+^ pipette solution containing (in mM) 130 K-gluconate, 5 KCI, 10 HEPES, 2 MgCI_2_, 0.5 EGTA, 2 ATP, 1 GTP (pH 7.3) was used in most experiments. Glass micropipettes were pulled with a horizontal puller (Sutter Instruments) using Sutter borosilicate glass capillaries with filament. The electrode resistance after back-filling was 2–4 MΩ. All voltages were corrected for the liquid junction potential (−13 mV).

### Chemicals

DPO-1 and TTX were from Tocris (Ellisville, MO), dendrotoxin-K, hongotoxin-1, margatoxin, were from Alomone Labs (Jerusalem, Israel), while the other substances were purchased from Sigma. The recording chamber was constantly perfused with aCSF (2–3 mL/min). The treatments were applied locally using a perfusion pencil system (tip diameter 100 µm, Automate Scientific) driven by gravity. Although we have not systematically constructed dose response curves, several concentrations were tested for each drug to assess its efficacy. After these trials we have chosen for each drug the concentration that produced the maximal effect. The doses used are similar with those reported in the literature.

### Temperature control

The temperature of the external solution was controlled with a TC-344B temperature controller and an inline heater (Warner Instruments, Hamden CT) and was maintained at 36–37°C.

### Data acquisition and analysis

**Figure 1:**
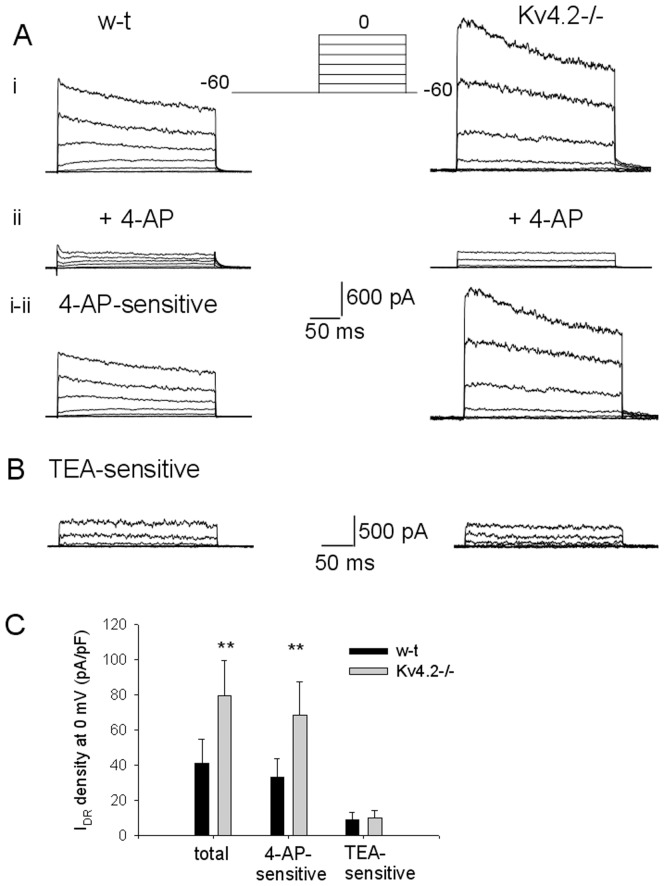
Delayed rectifier (I_DR_) K^+^ currents recorded in GAD65-GFP and Kv4.2−/−;GAD65-GFP preoptic GABAergic neurons and their sensitivities to 4-AP and TEA. A. Separation of the 4-AP-sensitive component (i–ii) of IDR in GAD65-GFP (left) and Kv4.2−/−;GAD65-GFP (right) neurons by subtraction of the currents measured in the presence of 4-AP (0.25 mM) (ii) from the control ones (i). IDR was elicited by the voltage step protocol depicted in the inset. Note the large IDR present in the Kv4.2−/−;GAD65-GFP neuron. TTX (1 µM) was present in all extracellular solutions. B. Representative TEA-sensitive component of IDR in a GAD65-GFP (left) and a Kv4.2−/−;GAD65-GFP (right) neuron. The TEA-sensitive component was separated by subtracting the currents measured in the presence of TEA (5 mM) from the control ones. The currents were elicited by the same voltage step protocol as in A. C. Bar chart summarizing the current density of the 4-AP -sensitive, TEA-sensitive and total IDR in GAD65-GFP and Kv4.2-/-;GAD65-GFP neurons. Bars represent averages +S.D. from n = 15 GAD65-GFP neurons and n = 19 Kv4.2−/−;GAD65-GFP neurons. The current densities were calculated using the IDR amplitude at the end of the depolarizing step to 0 mV. ** represents significant differences between GAD65-GFP and Kv4.2−/−;GAD65-GFP groups P<0.01 (t-test).

Data was acquired with a MultiClamp 700B amplifier digitized using a Digidata 1320A interface and the Pclamp9.2 software package (all from Molecular Devices, Sunnyvale, CA). The sampling rate for the continuous recordings of spontaneous activity was 50 kHz. The cell capacitance was determined and compensated using the Multiclamp Commander software. K^+^ currents were measured by step depolarizations to −60 mV to 0 mV in 10 mV increments from a holding potential of −60 mV (Fig 1A inset). The K^+^ currents were very stable over ∼30 min recordings in control extracellular solution displaying spontaneous changes of less than ±3%. The K^+^ currents characteristics were determined as previously described [6]. The spontaneous firing activity was measured in cell-attached configuration, voltage-clamp mode V_hold_ = 0 mV.

### RT/QPCR

Preoptic tissue obtained from slices was stored at −80 degree Celsius until the time of RNA extraction. Total RNA was extracted using Qiagen RNAeasy kit manufacturer's instructions. RNA extraction was conducted in dry ice. The concentration of RNA was determined using a nanodrop measuring the absorbance at 260 nm with reference to 280 nm. RNA was then stored at −80 degree Celsius. 4 µg RNA was then reverse-transcribed into first-complimentary DNA (cDNA) using Superscript III reverse transcriptase kit (Invitrogen, CA) according to manufacturer's instructions. The reaction was inactivated by incubating it at 75°C for 10 minutes. In order to remove RNA complimentary 1 µl RNAse H was added to the reaction and was incubated at 37°C for 20 minutes. The concentration of prepared cDNA was measured using nanodrop measuring absorbance at 230 nm with reference to 280 nm. Quantitative PCR reactions were performed in duplicate using Roche light-cycler and the following parameters were used for 45 cycles (95°C for 10 minutes, 95°C for 5 seconds, 60°C for 5 seconds, 72°C for 8 seconds for 45 cycles; for melting: 95°C for 5 seconds, 65°C for 5 seconds; for cooling 40°C for 30 seconds). Each 20 µl of PCR reaction contained: 4 µl Sybr green (Roche, enzyme mixed according to manufacturer's instructions), 9 µl PCR graded water, 2 µl 1 µM gene of interest primer and 5 µl diluted cDNA (1/30 dilution). Each reaction included a standard curve. To determine a standard curve, 1 µl of the non- diluted cDNA for all samples were mixed and a dilution of 1∶100, 1∶50 and 1: 25 was made. The standard curve is used for determining the efficiency of the primer and calculating the concentration of the mRNA amplified in the QPCR reaction. The concentration of each gene was normalized using the housekeeping gene GAPDH. Primers used were: Kv1.1 forward: TTACGAGTTGGGCGAGGA, reverse: TGACGATGGAGATGAGGATG; Kv1.3 forward: GCCGTGGTAACCATGACAACT, reverse: GCCCACAATCTTGCCTCCTA; Kv1.4 forward: ACGAGGGCTTTGTGAGAGA, reverse: TAAGATGACCAGGACGGACA; Kv1.5 forward: TGAGCATTCACTGTAAGATGG, reverse: TTGAGTTATCCCTCTGCTGGG; Kv2.1 forward: CACACAGCAATAGCGTTCAACTT, reverse: AGGCGTAGACACAGTTCGGC.

### Western blots

Acute slices containing the preoptic area were prepared as described above and the MnPO punched out. The tissue was then flash frozen in liquid nitrogen. Protein lysates were prepared using established methods and the the protein concentration in each sample was determined using a Bio-Rad protein assay kit. Equal amounts of proteins were fractionated on 7.5% SDS-PAGE gels using 7.5% Ready Gel Tris-HCl gels (BioRad, Hercules, CA). Electrotransfer of proteins from the gel to nitrocellulose membrane was performed for 60 min at 100 V. After incubation in blocking buffer at room temperature for 1 h, the membranes were incubated with primary antibodies against Kv1.5 subunits overnight at 4°C. The polyclonal Kv1.5 antibody (dilution 1∶500) was from Alomone labs (#APC-004). The specificity of the antibody was tested by the manufacturer as well as by previous studies [11], [12]. The blots were incubated in SuperSignal West Pico Chemiluminescent substrates (Thermo Scientific, Hanover Park, IL) for 5 min and visualized with autoradiography film (Denville Scientific, Metuchen, NJ). Blots were reprobed with primary antibodies against GAPDH (Abcam) to verify equal loading of lanes. For quantification, the anti-GAPDH signals were used to normalize the Kv1.5 subunit signals measured from the same blot. Quantification was performed using the National Institutes of Health Image J protocol.

### Statistics

The values reported are presented as mean + standard deviation (S.D.). Statistical significance of the results pooled from two groups was assessed with *t*-tests using Prism4 (GraphPad Software). Non-parametric one way analysis of variance (ANOVA, Kruskal-Wallis) with Dunn's post hoc test (*P*<0.05) was used for comparison of multiple groups.

## Results

### 4-AP-sensitive component of I_DR_ is specifically upregulated in preoptic GABAergic Kv4.2−/−;GAD65-GFP neurons

Based on previous observations that in preoptic GABAergic neurons the blockers 4-AP (0.25 mM) and TEA (5 mM) block distinct components of I_DR_ and that their co-application abolishes the current [7], [13] we have first evaluated them in GAD65-GFP and Kv4.2−/−;GAD65-GFP neurons. I_DR_ was activated from a holding potential of −60 mV to inactivate I_A_ [7]. The 4-AP sensitive component was significantly enhanced in Kv4.2−/−;GAD65-GFP neurons compared to GAD65-GFP neurons (Fig 1). Its density averaged 41±8 pA/pF (n = 15) and 79±11 pA/pF (n = 19) in GAD65-GFP and Kv4.2−/−;GAD65-GFP neurons, respectively (P<0.01, t-test). In contrast, the TEA-sensitive component was of similar amplitude in GAD65-GFP and Kv4.2−/−;GAD65-GFP mice (Fig 1). This indicates that the increased I_DR_ observed in Kv4.2−/− preoptic GABAergic neurons [7] is due to upregulation of 4-AP-sensitive Kv current, i.e. the Kv1 family [14].

**Figure 2:**
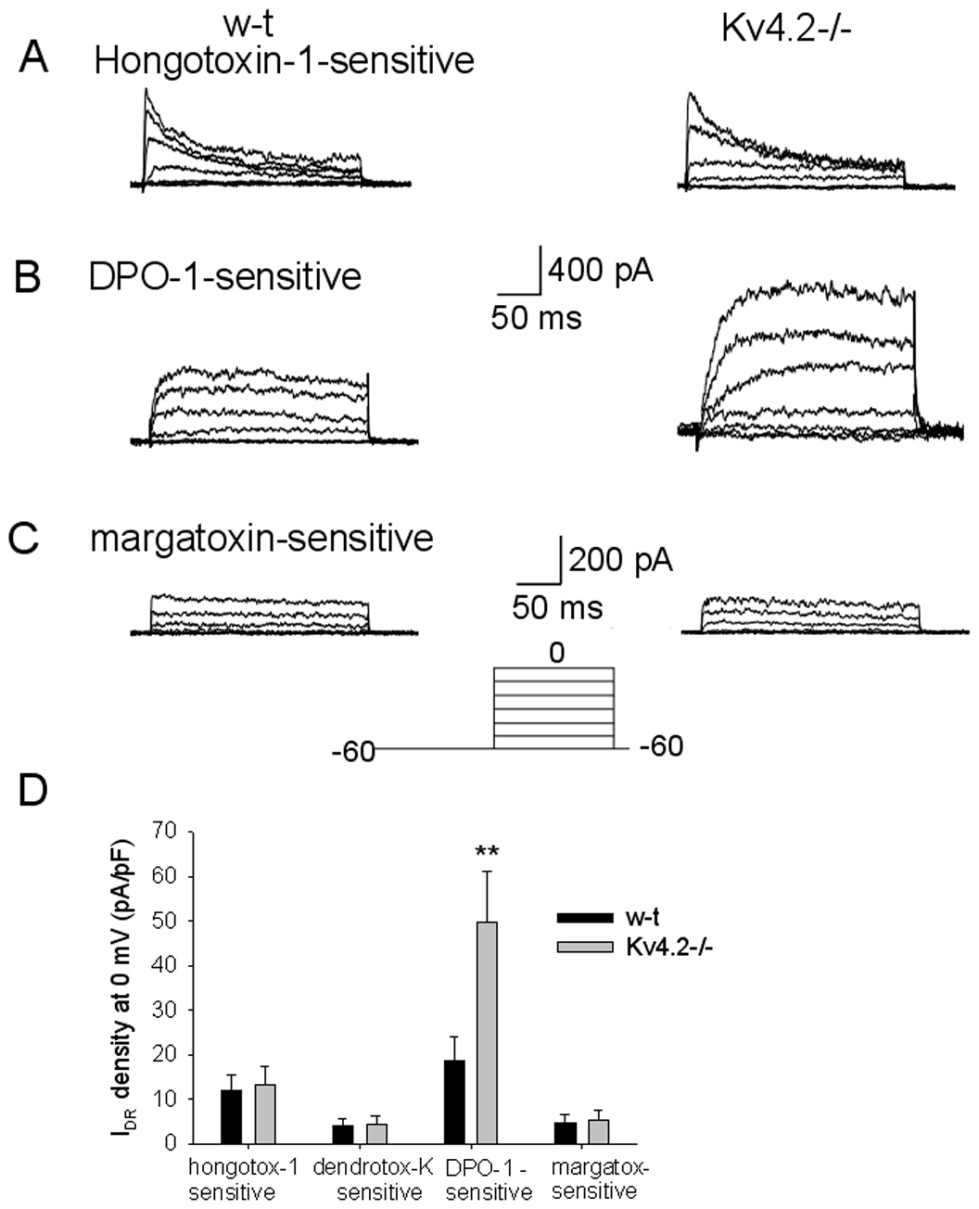
Differential sensitivity to K^+^ channel blockers of I_DR_ recorded in GAD65-GFP and Kv4.2−/−;GAD65-GFP preoptic GABAergic neurons. A. Hongotoxin-1 (0.3 nM) -sensitive component of I_DR_. Representative currents from a GAD65-GFP (left) and a Kv4.2−/−;GAD65-GFP (right) neuron. B. DPO-1 (0.5 µM) - sensitive component of I_DR_. Representative currents from a GAD65-GFP (left) and a Kv4.2−/−;GAD65-GFP (right) neuron. C. Margatoxin (5 nM) - sensitive component of I_DR_. Representative currents from a GAD65-GFP (left) and a Kv4.2−/−;GAD65-GFP (right) neuron. A-C. The currents were elicited by the same voltage step protocol depicted in the inset. TTX (1 µM) was present in all extracellular solutions. D. Bar chart summarizing the current density of the hongotoxin-1 (0.3 nM, n = 13 and n = 16 neurons, respectively)-sensitive, DPO-1 (0.5 µM, n = 23 and n = 26 neurons, respectively)-sensitive, dendrotoxin-K (50 nM, n = 5 and n = 6 neurons, respectively)-sensitive, and margatoxin (5 nM, n = 7 and n = 6 neurons, respectively)-sensitive in GAD65-GFP and Kv4.2−/−;GAD65-GFP neurons. Bars represent averages +S.D. The current densities were calculated using the I_DR_ amplitude at the end of the depolarizing step to 0 mV. ** represents significant differences between GAD65-GFP and Kv4.2−/−;GAD65-GFP groups P<0.01 (t-test).

We have then studied the actions of blockers selective for one or for several members of the Kv1 family to separate the respective components of I_DR_. Thus hongotoxin-1 (0.3 nM), a blocker of the Kv1.1, Kv1.2, Kv1.3 and Kv1.6 subunits [15], blocked a current of similar amplitude in GAD65-GFP and Kv4.2−/−;GAD65-GFP neurons (Fig 2). The hongotoxin-1-sensitive component averaged 11±3 pA/pF (n = 13) and 12±4 pA/pF (n = 16) in GAD65-GFP and Kv4.2−/−;GAD65-GFP neurons, respectively (P>0.01, t-test). Similarly, dendrotoxin-K (50 nM) and margatoxin (5 nM), selective blockers for Kv1.1 and Kv1.3 [16], [17], respectively, abolished currents of comparable densities in GAD∧%-GFP and Kv4.2−/−;GAD65-GFP neurons. The dendrotoxin-K -sensitive components were 3±1 pA/pF (n = 5) and 4±2 pA/pF (n = 6) in GAD65-GFP and Kv4.2−/−;GAD65-GFP neurons, respectively (P>0.01, t-test), while the margatoxin-sensitive components were 5±2 pA/pF (n = 7) and 6±3 pA/pF (n = 6) in GAD65-GFP and Kv4.2−/−;GAD65-GFP neurons, respectively (P>0.01, t-test).

In contrast, I_DR_ of Kv4.2−/−;GAD65-GFP neurons was much more sensitive than that of GAD65-GFP neurons to the Kv1.5 selective blocker DPO-1 (0.5 µM) [18](Fig 2). The DPO-1 -sensitive component averaged 18±6 pA/pF (n = 23) and 51±12 pA/pF (n = 26) in GAD65-GFP and Kv4.2−/−;GAD65-GFP neurons, respectively (P<0.01, t-test). The same result was obtained with a different Kv1.5 selective blocker, mephetyl tetrazole (0.6 µM) [19]. The component blocked by the drug averaged 16±5 pA/pF (n = 4) and 49±11 pA/pF (n = 5) in GAD65-GFP and Kv4.2−/−;GAD65-GFP neurons, respectively (P<0.01, t-test). Interestingly, the increase in the components sensitive to the two Kv1.5 blockers (∼33 pA/PF) could account for the increase in total I_DR_ measured in Kv4.2−/−;GAD65-GFP neurons, that averaged ∼38 pA/pF (Fig 1).

These experiments also revealed, surprisingly, that the DPO-1-sensitive component had strikingly slower kinetics of activation when compared to the other components (Table 1).

**Table 1:** Properties of the I_DR_ components.

I_DR_ component		Threshold of activation (mV)	Time to peak at 0 mV test potential (ms)	% decay at the end of 250 ms step to 0 mV
**DPO-1- sensitive I_DR_**	GAD65-GFP	−35±4 (23)	24±8 (23)	18±7 (23)
	Kv4.2−/−;GAD65-GFP	−33±5(26)	23±7 (26)	14±4 (26)
**Margatoxin- sensitive I_DR_**	GAD65-GFP	−36±4(7)	5±2 (7)	6±4 (7)
	Kv4.2−/−;GAD65-GFP	−32±5(6)	6±3 (6)	8±5 (6)
**Dendrotoxin-k- sensitive I_DR_**	GAD65-GFP	−35±4(5)	5±1 (5)	6±4 (5)
	Kv4.2−/−;GAD65-GFP	−34±3(6)	5±2 (6)	8±5 (6)
**Hongotoxin-1- sensitive I_DR_**	GAD65-GFP	−36±5(13)	6±2 (13)	68±19 (13)
	Kv4.2−/−;GAD65-GFP	−34±6(16)	7±3 (16)	54±15 (16)
**TEA - sensitive I_DR_**	GAD65-GFP	−18±4(7)	14±6 (7)	4±3 (7)
	Kv4.2−/−;GAD65-GFP	−20±3(8)	15±7 (8)	7±4 (8)

Values are presented as mean ±S.D., (n)-number of cells.

**Figure 3:**
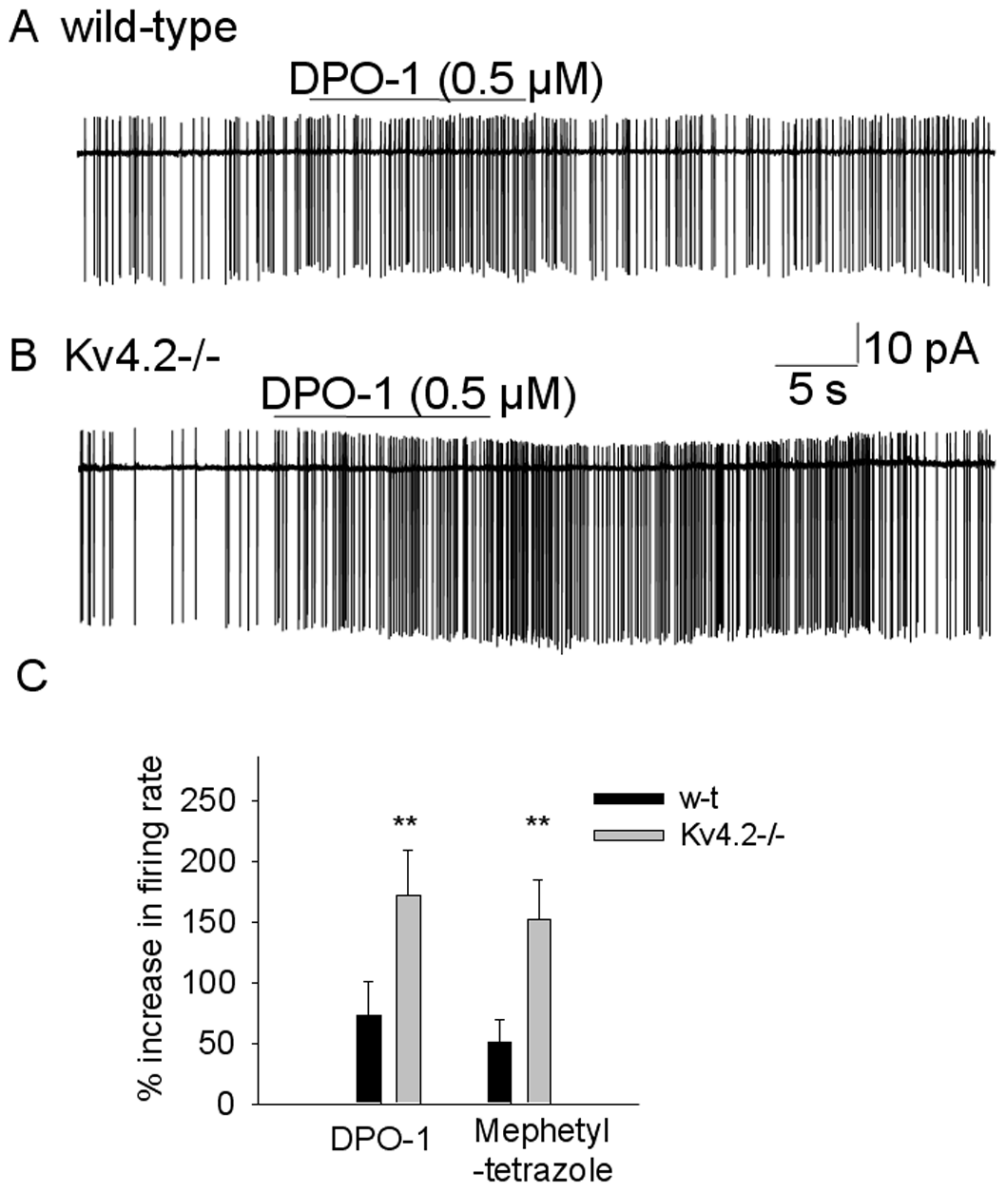
Effects on DPO-1 on the spontaneous firing activity of GABAergic preoptic neurons from GAD65-GFP and Kv4.2−/−;GAD65-GFP mice. A. Cell-attached recordings showing spontaneous firing activity in control, during DPO-1 (0.5 µM) application, and during washout from a GAD65-GFP (A) and a Kv4.2−/−;GAD65-GFP (B) GABAergic neuron. C. Bar chart summarizing the maximum effect of DPO-1 (0.5 µM) and mephetyl tetrazole (0.5 µM) on the firing rate of GABAergic preoptic neurons from GAD65-GFP (n = 8 and n = 5 neurons, respectively) and Kv4.2−/−;GAD65-GFP mice (n = 12 and n = 7 neurons, respectively). ** represents significant differences between GAD65-GFPand Kv4.2−/−;GAD65-GFP groups P<0.01 (ANOVA).

To assess the functional role of the Kv1.5 subunits in preoptic GABAergic neurons we measured the effect of the Kv1.5 blockers on their spontaneous firing activity. DPO-1 (0.5 µM) increased the spontaneous firing rate from 8.9±3.1 Hz to 15.2±5.4 Hz in GAD65-GFP neurons (n = 8)(Fig 3A). The drug had significantly larger effect in Kv4.2−/−;GAD65-GFP neurons where it increased the firing rate from 8.5±2.6 Hz to 22.9±7.1 Hz (n = 12). Similarly, mephetyl tetrazole (0.6 µM) increased the firing rate from 9.1±3.7 Hz to 13.3±4.2 Hz in GAD65-GFPneurons (n = 5) and from 8.2±2.5 Hz to 20.5±5.9 Hz in Kv4.2−/−;GAD65-GFP neurons (n = 7). Both drugs were more efficacious in Kv4.2−/−;GAD65-GFP neurons (P<0.01, ANOVA) (Fig 3B).

### Molecular identity of the K^+^ channels conducting 4-AP sensitive I_DR_ in Kv4.2−/−;GAD65-GFP preoptic neurons

**Figure 4:**
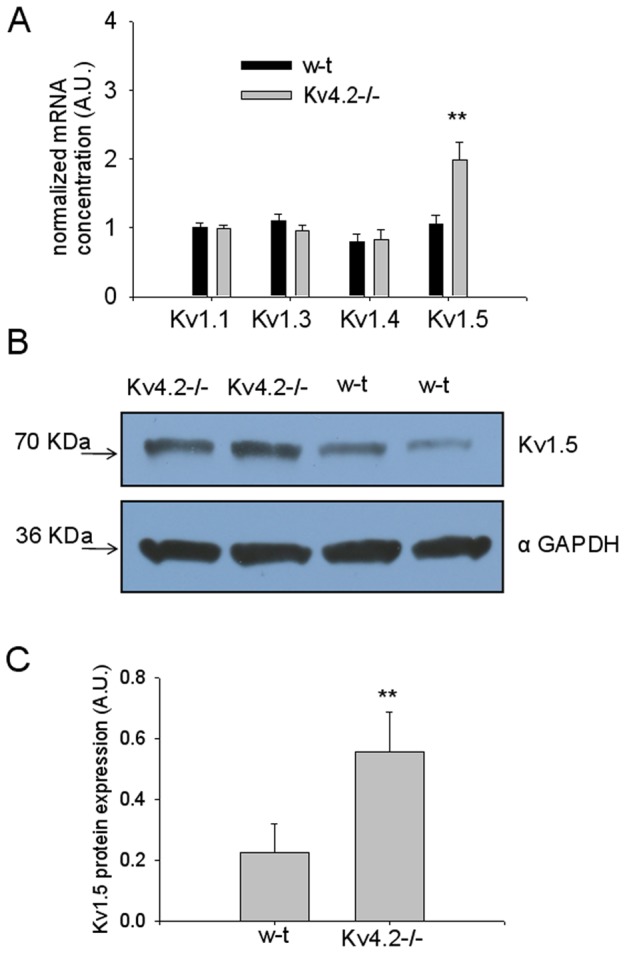
Upregulation of the level of expression of Kv1.5 subunits in Kv4.2−/−;GAD65-GFP preoptic tissue. A. qPCR analysis of Kv1.1, 1.3, 1.4 and 1.5 expression in preoptic area from GAD65-GFP and Kv4.2−/−;GAD65-GFP mice (n = 6 mice each). For normalization GAPDH was selected as housekeeping gene. Bars represent averaged normalized concentrations ±SD. ** represents significant differences between GAD65-GFP and Kv4.2−/−;GAD65-GFP groups P<0.01 (t-test). B. Western blot analysis of the expression of Kv1.5 subunits. (A) Lysates prepared from the preoptic area of GAD65-GFP or Kv4.2−/−;GAD65-GFP mice (n = 6 animals for each genotype) were fractionated, transferred to PVDF membranes and probed with a specific anti-Kv1.5 antibody. Blots were also probed with antibodies against GAPDH to confirm equal loading of proteins. In each lane, anti-Kv1.5 antibody signals were quantified and normalized to the anti-GAPDH antibody signals. C. In Kv4.2−/−;GAD65-GFP preoptic area, the mean ±S.D. expression levels of Kv1.5 protein were significantly (**, p<0.01) higher than in GAD65-GFP preoptic area. Molecular masses are indicated on the blots by arrows.

To clarify the expression of the Kv subunits we have performed qPCR on RNA extracted from preoptic tissue from GAD65-GFP and Kv4.2−/−;GAD65-GFP mice (n = 6 mice each). After reverse transcription of RNA was performed, Kv1.1, Kv1.3, Kv1.4, Kv1.5 and GAPDH expression was measured using qPCR (Fig 1A). When normalized with respect to GAPDH gene expression, levels of Kv1.5 were found to be ∼90% increased in the Kv4.2−/−;GAD65-GFP samples when compared to the wild-type samples (t-test, P<0.01). In contrast, the levels for Kv1.1, Kv1.3, and Kv1.4 transcripts were similar (t-test, P>0.2) (Fig 4A). The expression of the Kv2.1 subunits was also similarly quantified and no differences between GAD65-GFP and Kv4.2−/−;GAD65-GFP tissues (data not shown) were found.

Western blot analysis of preoptic tissue lysates was then performed to assess the expression of Kv1.5 protein. Representative Western blots of total protein from GAD65-GFP and Kv4.2−/−;GAD65-GFP preoptic tissue (n = 6 mice each) probed with a Kv1.5 antibody are presented in Fig 4B. The expression level of Kv1.5 protein was 140% higher than that measured in the GAD65-GFP mice (Fig 4B,C)

## Discussion

This data clarifies the molecular nature of the upregulated I_DR_ in preoptic GABAergic neurons of Kv4.2−/−;GAD65-GFP mice. Using selective blockers, qPCR and Western blots we identify Kv1.5 as the subunit responsible for the bulk of the upregulated I_DR_ component. Thus, Kv4.2−/−;GAD65-GFP preoptic neurons, present both upregulation of Kv4.1 [7], a subunit from the same family, as well as upregulation of Kv1.5, a subunit with differential biophysical characteristics. We also show that Kv1.5 upregulation is functionally relevant: blocking the respective current results in robust enhancement of the spontaneous firing rate, effect much more pronounced in Kv4.2−/−;GAD65-GFP neurons. Surprisingly, the DPO-1- sensitive current displayed slow kinetics, situation opposite to observations in heart muscle, where the drug blocks the ultrarapid I_DR_ [18]. Thus it appears that Kv1.5 subunits form channels with differential characteristics in preoptic neurons and heart muscle, respectively. A possibility that remains to be investigated is that different accessory subunits modulate Kv1.5 in the two tissues. The slow kinetics of activation of the current could also be explained by the presence of Kv1.5-Kv1.2 heterotetramers, as found in vascular myocytes [20]. Because of their slow gating kinetics it is likely that the currents conducted by Kv1.5-containing channels play a role in the afterhyperpolarization phase of the action potential. A larger and slower afterhyperpolarization would prolong the interspike interval.

This study also provides, for the first time, information regarding the pharmacological and molecular characteristics of I_DR_ in GAD65-GFP preoptic GABAergic neurons. The majority of the current (∼80%) is conducted by subunits of the Kv1 family since it is sensitive to low 4-AP concentrations [14]. Using selective blockers we have separated components conducted by Kv1.5, Kv1.3 and Kv1.1-containing channels. Interestingly, we have revealed that the Kv1.5-containing channels have slow gating kinetics, while the hongotoxin-1- sensitive component activated rapidly and decayed significantly during the depolarizing step. Since the pharmacologically-isolated Kv1.1 and Kv1.3 components did not display such inactivation it is likely that Kv1.2 or Kv1.6 are also involved in this current. Although their sensitivity to hongotoxin-1 is not known, Kv1.4 subunits could also be responsible for the decaying phase of the current [21]. As previously reported the remaining I_DR_ (∼20%) is blocked by 5 mM TEA [7]. This current displayed little decay and activated at potential more depolarized (∼10–20 mV) than the 4-AP- sensitive component. Taken together these properties are reminiscent of those of the Kv2 family [14]. Using qPCR we have detected Kv2.1 transcripts, however the expression of other Kv2 subunits cannot be ruled out.

In this study we reveal a novel mechanism of electrical remodeling in a central neuron in response to genetic deletion of a channel subunit. The mechanisms by which a neuron selects the means by which to compensate for the lack of a channel subunit are not known, nevertheless by identifying the effector subunits involved we provide here a first step. Their understanding may yield novel therapeutic strategies for reversing electrical remodeling relevant to pathologies.
